# *In vivo* targeting of miR‐223 in experimental eosinophilic oesophagitis

**DOI:** 10.1002/cti2.1210

**Published:** 2020-11-23

**Authors:** Adam M Collison, Leon A Sokulsky, Scott Nightingale, Elizabeth Percival, Anna LeFevre, Joseph Meredith, Sybille Krauss, Paul S Foster, Joerg Mattes

**Affiliations:** ^1^ Experimental and Translational Respiratory Medicine Group Newcastle NSW Australia; ^2^ Priority Research Centre GrowUpWell The University of Newcastle and Hunter Medical Research Institute Newcastle NSW Australia; ^3^ Priority Research Centre for Healthy Lungs The University of Newcastle and Hunter Medical Research Institute Newcastle NSW Australia; ^4^ Paediatric Gastroenterology Department John Hunter Children's Hospital Newcastle NSW Australia; ^5^ Faculty IV: School of Science and Technology Institute of Biology Department Human Biology/Neurobiology University of Siegen Siegen Germany; ^6^ Paediatric Respiratory & Sleep Medicine Department Newcastle Children's Hospital Kaleidoscope Newcastle NSW Australia

**Keywords:** eosinophilic oesophagitis, microRNA, Midline‐1, resveratrol

## Abstract

**Objectives:**

Eosinophilic oesophagitis (EoE) is characterised by oesophageal inflammation, fibrosis and dysfunction. Micro (mi)‐RNAs interfere with pro‐inflammatory and pro‐fibrotic transcriptional programs, and miR‐223 was upregulated in oesophageal mucosal biopsy specimens from EoE patients. The therapeutic potential of modulating miR‐223 expression *in vivo* has not been determined. We aimed to elucidate the relevance of oesophageal miR‐223 expression in an *in vivo* model of EoE by inhibiting miR‐223 tissue expression.

**Methods:**

The expression of miR‐223 and the validated miR‐223 target insulin‐like growth factor receptor 1 (IGF1R) protein was determined in our paediatric cohort of EoE patients. A murine model of *Aspergillus fumigatus*‐induced EoE was employed, and oesophagi were assessed for miR‐233, IGF1R, T lymphocyte type 2 (T2) cytokine expression and eosinophil infiltration. Mice were treated with antagomirs targeting miR‐223 or resveratrol targeting its upstream regulator Midline‐1(MID‐1).

**Results:**

There was an inverse relationship between an increased expression of miR‐223 and a decreased IGF1R protein concentration in biopsy specimens from EoE patients. TNF‐related apoptosis‐inducing ligand deficiency, MID‐1 inhibition and resveratrol treatment suppressed miR‐223 expression. Furthermore, inhibition of miR‐223 and treatment with resveratrol in the oesophagus resulted in an amelioration of EoE hallmark features including eosinophilic infiltration, oesophageal circumference and a reduction in T2 cytokine expression.

**Conclusion:**

miR‐223 has a key role in the perpetuation of EoE hallmark features downstream of TNF‐related apoptosis‐inducing ligand and MID‐1 in an experimental model. These studies highlight a potentially critical role of miRNA function in EoE aetiology. miR‐223 expression in the oesophagus may be therapeutically modulated by resveratrol, providing a potential new therapeutic option to be explored in EoE patients for this increasingly prevalent condition.

## Graphical abstract

miR‐223 has a key role in the perpetuation of eosinophilic oesophagitis hallmark features downstream of TNF‐related apoptosis‐inducing ligand and MID‐1. These studies highlight a critical role of miRNA function in eosinophilic oesophagitis aetiology and suggest that miR‐223 expression in the oesophagus can be therapeutically modulated by resveratrol, providing a potential new therapy for this increasingly prevalent condition with limited current treatment options.

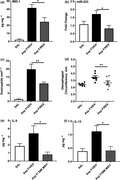

## Introduction

Eosinophilic oesophagitis (EoE) is a chronic inflammatory disorder characterised by symptoms of oesophageal dysfunction and eosinophilic infiltration of the oesophagus,^1^ with rising incidence and prevalence.^2^ There is an increasing body of evidence that suggests that EoE is driven by food and aero‐allergen exposure,^3^, ^4^, ^5^ resulting in the activation of interleukins (IL)‐5, IL‐13 and IL‐33, subsequently promoting eosinophil migration into and activation in the oesophagus, ultimately leading to oesophageal remodelling.^4^, ^6^, ^7^ Current therapeutic options are limited to long‐term use of proton pump inhibitors, swallowed corticosteroids, elimination diets or oesophageal balloon dilatation, which offer varying success and risks of adverse effects or negative impact on quality of life.^8^ There is a need for new, effective and safe therapies for EoE.

Analysis of the EoE transcriptome suggests that, in addition to the dysregulation of EoE associated genes, there could be a significant role for non‐coding RNA influence in the pathogenesis of EoE in the form of long‐coding RNAs and micro (mi)‐RNAs.^9^, ^10^ miRNAs belong to a class of interfering RNAs, typically 19–25 nucleotide bases in length, responsible for the manipulation of gene translation.^11^ Epigenetic modulation of such genes occurs through the formation of the RNA‐induced silencing complex (RISC), which will bind to target mRNAs resulting in degradation of the target mRNA sequence.^12^, ^13^ Experimentally, specific miRNAs can be inhibited with cholesterol‐conjugated complimentary sequences known as ‘antagomirs’, which have been used to highlight key miRNA‐mRNA interactions in disorders such as allergic asthma.^14^, ^15^, ^16^ miRNA array analysis of EoE has demonstrated a profile of miRNAs that could be in the perpetuation of EoE‐associated inflammation and remodelling. A miRNA signature was observed in EoE patients consisting of an upregulation of miR‐21, miR‐223, miR‐206 and let‐7, as well as a downregulation of miR‐375.^10^ While such studies provided key insights on the epigenetic miRNA signature of EoE patients, the effect of miRNA modulation on the EoE phenotype has not been thoroughly investigated.

Our previous study focusing on TNF‐related apoptosis‐inducing ligand (TRAIL) signalling in EoE employed silencing (si)‐RNA technology to downregulate the expression of Midline (MID)‐1 in the oesophagi of *Aspergillus fumigatus* (*A. fumigatus*)‐challenged mice.^7^ Here, we demonstrate a functional effect of miR‐223 in an *A. fumigatus* model of EoE in regard to eosinophilic inflammation, remodelling and T2 cytokine expression. Recently, the stilbenoid resveratrol has identified as a direct small molecule inhibitor of MID‐1.^17^ Here – for the first time – we exploit this novel property to inhibit MID‐1 protein *in vivo* upstream of miR‐223 in the oesophagus to ameliorate EoE hallmark features.

## Results

### miR‐223 is upregulated in EoE patient tissue and inversely correlated with IGF1R

**Figure 1:**
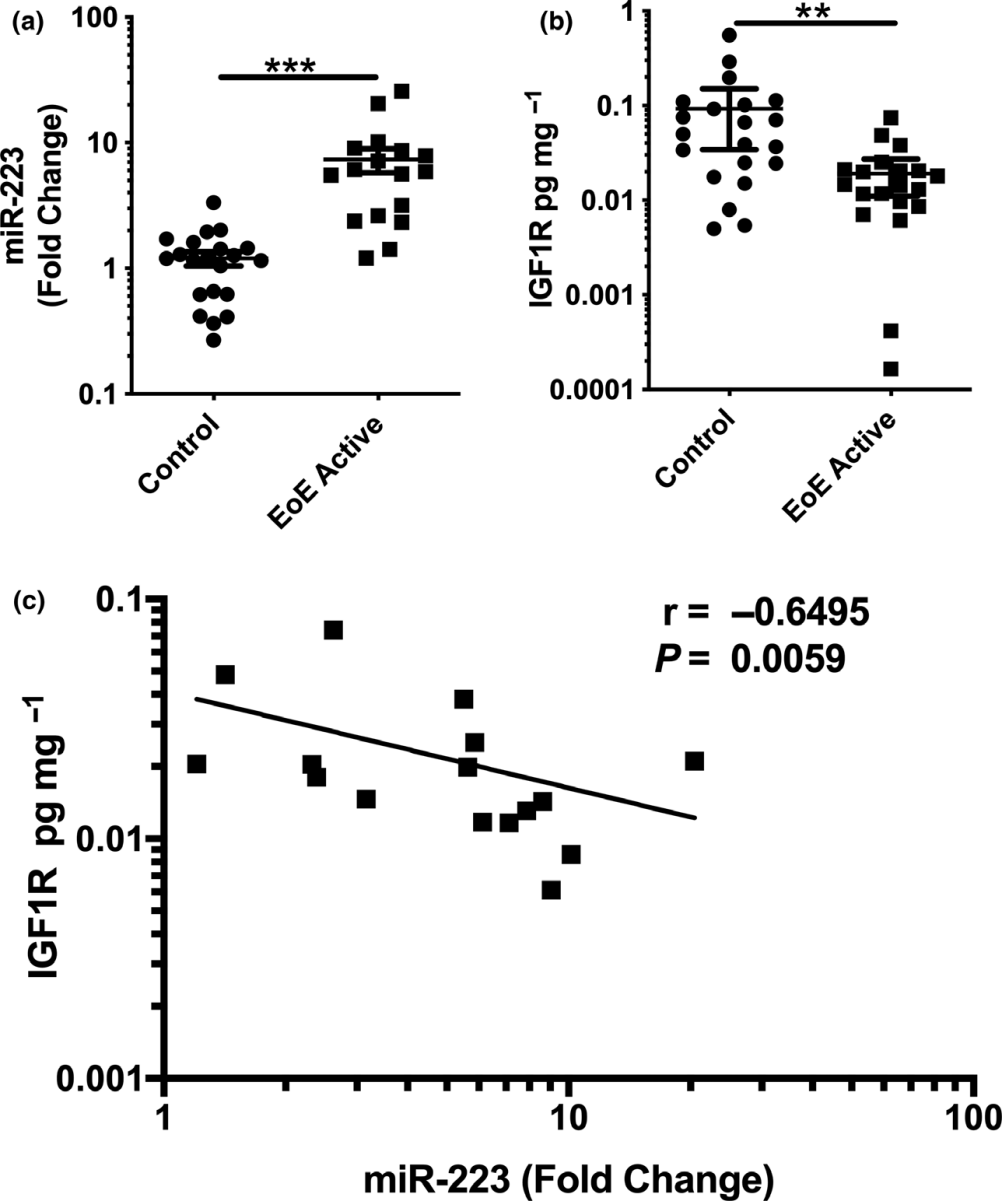
miR‐223 is upregulated in eosinophilic oesophagitis (EoE) patients and is inversely correlated with IGF1R protein expression. **(a)** Relative expression of miR‐223 in EoE patients (*n* = 17) normalised to U6 and healthy controls (*n* = 21) determined by TaqMan™ qPCR. **(b)** IGF1R protein as quantified by ELISA is downregulated in biopsies from EoE patients compared to control biopsies. Statistical significance was determined using Mann–Whitney analysis. **(c)** IGF1R protein correlated with miR‐223 expression in EoE and patient biopsies (*n* = 17). Statistical significance was determined using Spearman's correlations (*r*). Data are from a single technical replicate experiment, expressed as mean ± SEM. **P < 0.01, ***P < 0.001.

RNA extracted from oesophageal biopsies, from children histologically confirmed to have active EoE and subjects that were controls, was used to determine the expression of miR‐223 in our paediatric EoE cohort through the use of TaqMan™ RT and qPCR primers. Supporting published findings,^18^ miR‐223 was found to be significantly upregulated in EoE‐active patients when compared to controls (Figure 1a).

Additionally, we explored the expression of the previously confirmed miR‐223 target insulin‐like growth factor receptor 1 (IGF1R) in patient biopsy samples. We found reduced levels of IGF1R when compared to controls (Figure 1b) and the expression of miR‐223 inversely correlated with IGF1R in confirmed active EoE cases (Figure 1c).

### Upregulated miR‐223 in *A. fumigatus‐*exposed murine oesophagi is associated with IGF1R, TRAIL and MID‐1

**Figure 2:**
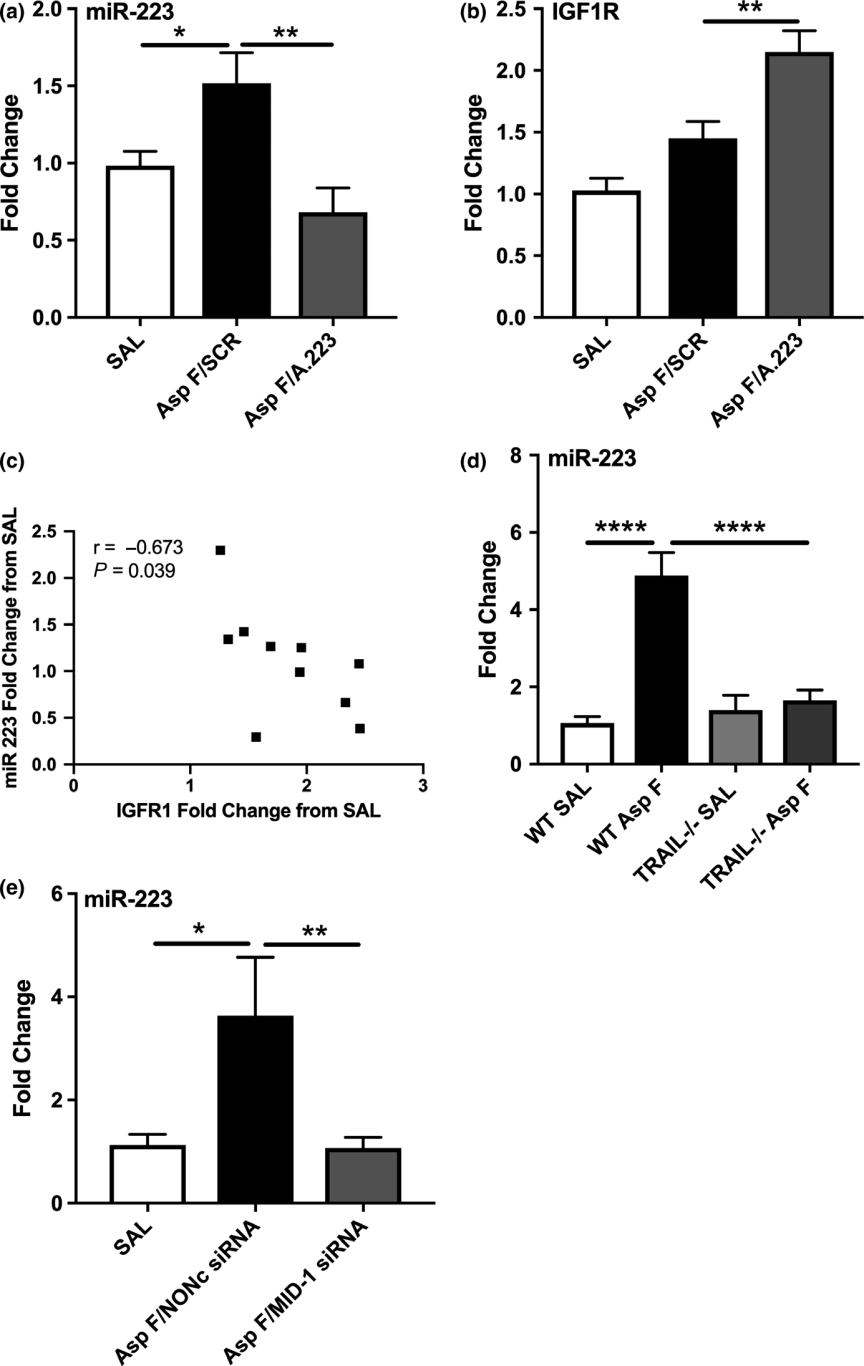
miR‐223 expression is upregulated in *Aspergillus fumigatus* (Asp F)‐exposed murine oesophagi, is associated with IGF1R downregulation and is downstream of TNF‐related apoptosis‐inducing ligand (TRAIL) and MID‐1. Relative expression of oesophageal miR‐223 *in vivo* in **(a)** wild‐type BALB/c mice treated with scrambled (SCR) antagomir or antagomir targeting miR‐223 (A.223). **(b)** Expression of IGF1R in BALB/c oesophagi exposed to *A. fumigatus* and treated with SCR or A.223. **(c)** Spearman's correlation of oesophageal miR‐223 and IGF1R in BALB/c mice. **(d, e)** miR‐223 expression of **(d)** *A. fumigatus*‐exposed wild‐type BALB/c (WT) and TRAIL‐deficient^−/−^ mice on a BALB/c background and **(e)** wild‐type BALB/c mice treated with *A. fumigatus* and administered either nonsense scrambled control sequence (NONc) siRNA or siRNA targeting Midline‐1 (MID‐1). Expression is normalised to Sno202 and saline (SAL) control group for miR‐223, and β‐actin and SAL control group for IGF1R (*n* = 5–7). Data are from a single technical replicate experiment, expressed as mean ± SEM. *P < 0.05, **P < 0.01, ****P < 0.0001.

We continued to explore the role of miR‐223 by employing an *in vivo* model of *A. fumigatus*‐driven EoE. Mice exposed to *A. fumigatus* demonstrated a significant increase in miR‐223 expression, which was successfully knocked down to baseline levels upon intranasal administrations of antagomir targeting miR‐223 compared to mice that received a scrambled control (SCR) antagomir (Figure 2a). Following miR‐223 knockdown with antagomir treatment, IGF1R expression was increased compared to SCR control‐treated mice (Figure 2b). As in the patient samples, there was a negative correlation between miR‐223 and IGF1R in *A. fumigatus‐*sensitised and challenged mice (Figure 2c).

TRAIL‐deficient mice have previously been shown to be largely protected from the EoE phenotype.^7^ Gene expression analysis of WT and TRAIL^−/−^ mice exposed to *A. fumigatus* revealed that TRAIL deficiency was associated with reduced levels of miR‐223 expression in the oesophagus (Figure 2d). Likewise, mice intranasally treated with an siRNA targeting MID‐1 – that has a pro‐inflammatory signalling role downstream of TRAIL^7^, ^19^ – also reduced the expression of miR‐223 in the oesophagus of *A. fumigatus*‐treated mice (Figure 2e).

### Eosinophilia, remodelling and T2 cytokine expression are dependent on miR‐223 expression in *A. fumigatus‐*driven EoE

**Figure 3:**
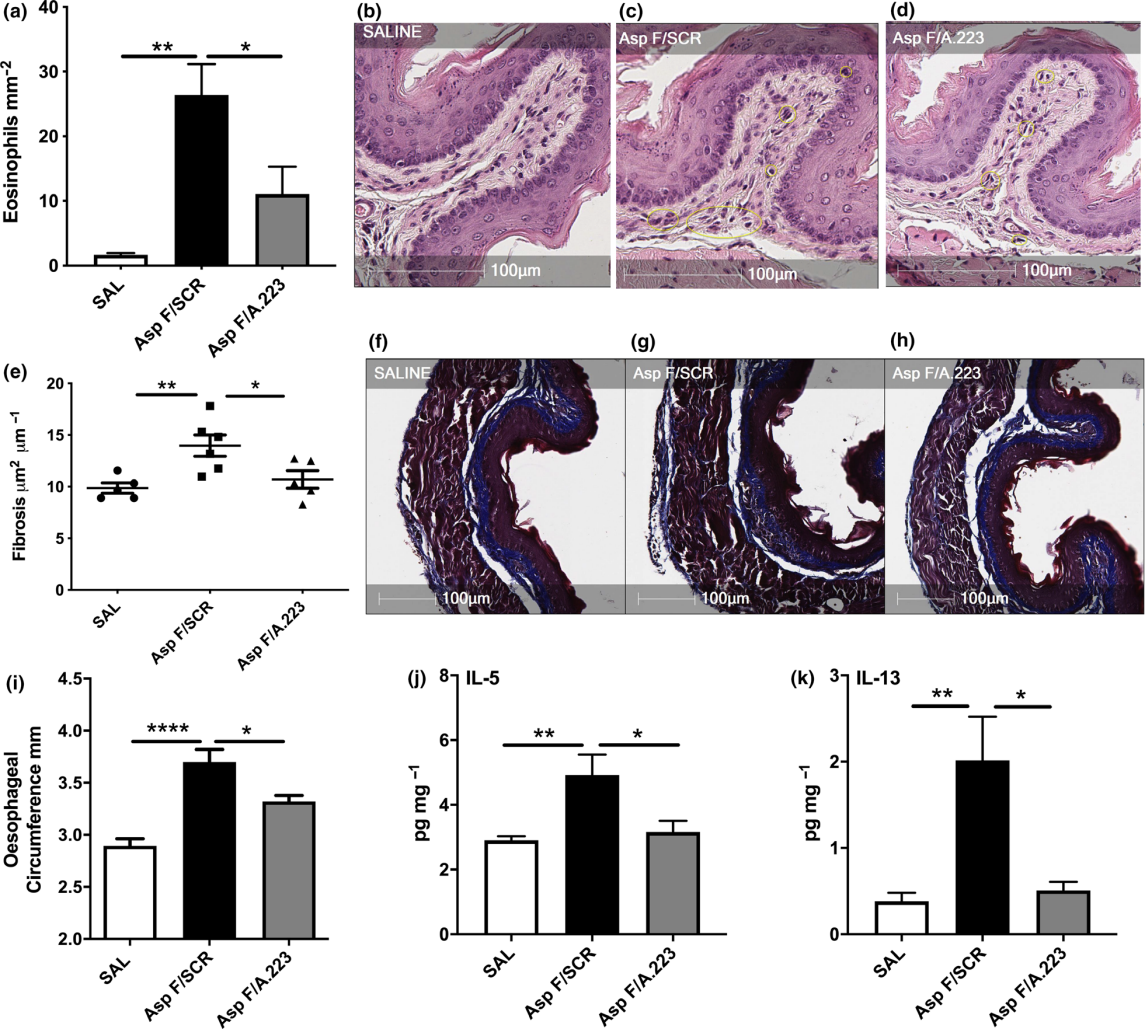
Inhibition of miR‐223 in the oesophagus results in reduced eosinophilic infiltration of the oesophagus and oesophageal enlargement with antagomir‐223. **(a)** Enumeration of eosinophils present in the oesophageal squamous‐epithelial cell layer as determined by light microscopy analysis of H&E‐stained BALB/c oesophageal transverse sections (*n* = 4 or 5).**(b–d)**Representative images of **(b)** SAL, **(c)** AspF/SCR or **(d)** AspF/A.223 with eosinophils circled in yellow. **(e)** Oesophageal fibrosis analysis with representative images of **(f)** SAL, **(g)** AspF/Scr and **(h)** AspF/A223‐treated BALB/c mice. **(i)** Oesophageal circumference analysis of oesophageal tissue determined using image analysis (*n* = 5–8). **(j, k)** Protein expression of **(j)** IL‐5 and **(k)** IL‐13 determined by ELISA and normalised to tissue BSA (*n* = 5–8). Data are from a single technical replicate experiment, expressed as mean ± SEM. *P < 0.05, **P < 0.01, ****P < 0.001.

Next, the impact miR‐223 on eosinophilic inflammation of the oesophagus was assessed through the enumeration of eosinophil infiltrates in transverse, H&E‐stained oesophageal sections under light microscopy (Figure 3a). As expected, *A. fumigatus*‐exposed mice given scrambled control antagomir had an elevated number of eosinophils within the squamous‐epithelial region of the oesophagus. In mice that were receiving antagomir‐223 therapy, however, there was a marked reduction of eosinophil infiltration when compared to the scrambled control group (Figure 3a–d).

We then assessed the impact of miR‐223 inhibition on key remodelling features seen in murine EoE, subepithelial fibrosis and oesophageal enlargement. Performing image analysis, we found that miR‐223 antagomir treatment reduced *A. fumigatus*‐induced subepithelial fibrosis (Figure 3e–h) and enlargement of the oesophageal circumference when compared to the appropriate controls (Figure 3i).

To further understand the pro‐inflammatory role of miR‐223 in *A. fumigatus*‐driven EoE, the eosinophil‐associated T2 cytokines (IL‐5 and IL‐13) were assessed in protein‐extracted oesophageal homogenates through the use of ELISA. Here, we demonstrate that mice with diminished miR‐223 expression through antagomir‐223 had a reduction of IL‐5 and IL‐13 protein expression despite *A. fumigatus* exposure (Figure 3j and k).

### Administration of resveratrol in *A. fumigatus‐*driven EoE results in both miR‐223 downregulation in addition to EoE inflammatory features

**Figure 4:**
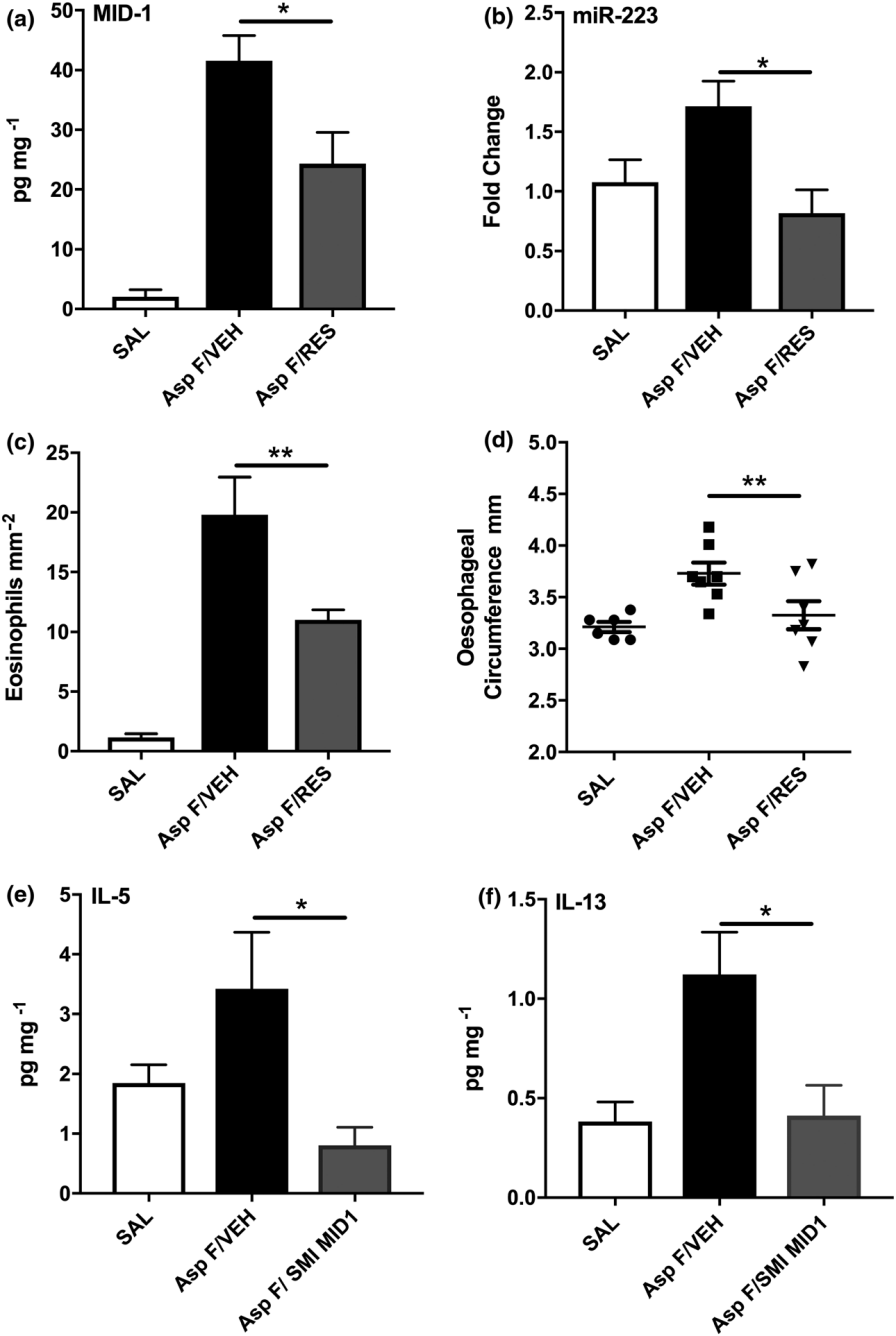
Resveratrol reduces MID‐1 and miR‐223 expression while reducing eosinophilic oesophagitis (EoE) features. **(a)** The E3 ubiquitin ligase Midline‐1 (MID‐1) is upregulated in *Aspergillus fumigatus* (Asp F)‐exposed C57Bl/6 mice and inhibited by oral resveratrol (RES; *n* = 3–6). **(b)** Resveratrol treatment decreased miR‐223 expression (*n* = 5 or 6). **(c)** Enumeration of eosinophils present in the oesophageal squamous‐epithelial cell layer (per mm^2^) as determined by light microscopy analysis of H&E‐stained C57Bl/6 oesophageal transverse sections (*n* = 6 or 7). **(d)** Oesophageal circumference analysis of oesophageal tissue determined using image analysis (*n* = 6 or 7) in mice treated with RES. **(e, f)** Protein expression of **(e)** IL‐5 and **(f)** IL‐13 determined by ELISA and normalised to tissue BSA (*n* = 5 or 6). Data are from a single technical replicate experiment, expressed as mean ± SEM. *P < 0.05, **P < 0.01.

Oral administration of the stilbenoid resveratrol (RES) throughout the model effectively reduced the expression of MID1 in the oesophagus from 24hrs following *A. fumigatus* administration (Figure 4a). Additionally, we determined that resveratrol treatment effectively reduced the expression of oesophageal miR‐223 (Figure 4b), similar to what occurs in MID‐1 siRNA‐treated mice. We further explored the ability of resveratrol to modulate EoE through MID‐1/miR‐223 by exploring disease features previously investigated. Here, we demonstrate that resveratrol reduces eosinophilic inflammation (Figure 4c), oesophageal enlargement (Figure 4d) and oesophageal expression of IL‐5 (Figure 4e) and IL‐13 (Figure 4f), similar to what occurs in antagomir‐223‐treated mice.

## Discussion

It has been established that dysregulation of miRNA occurs in the oesophagus of EoE patients; however, until now there have not been any studies that have investigated the function of specific miRNAs and the consequences of inhibiting miRNAs of interest either directly such as with antagomirs, or through blocking of upstream pathways thought to be associated with miRNA expression. miR‐223 is one of the most upregulated miRNAs found in EoE patient biopsies, which has been demonstrated in multiple previous studies^10^, ^18^ in addition to our cohort of EoE paediatric patients. Here, we further explore the role of this miRNA through *in vivo* studies, where we demonstrate for the first time that antagomir targeting therapy in the oesophagus can effectively downregulate the expression of miR‐223 and reduce hallmark features associated with EoE. This study is the first of its kind to highlight the therapeutic effectiveness of silencing miRNA and their upstream signalling pathways in the oesophagus in mouse models of EoE.

Inhibition of miR‐223 resulted in a reduction of the T2 cytokines IL‐5 and IL‐13. Both cytokines have been shown to play an essential role in the perpetuation of EoE, with IL‐5 being involved in eosinophil maturation and recruitment to the oesophagus while IL‐13 elicits local allergenic effects and the activation of the STAT6 pathway.^20^, ^21^

Hallmark histological EoE features of inflammation and remodelling were measured in antagomir‐ and resveratrol‐treated mice challenged with *A. fumigatus*. It was found that miR‐223 promoted eosinophilic infiltration in the oesophagus and increased oesophageal circumference and subepithelial fibrosis. While the clinical relevance of the observed reduction in eosinophilic inflammation remains elusive, these results provide evidence for the proposal that miR‐223 inhibition or associated pathways may be a potential therapeutic option for EoE. Given the regulatory effect of miR‐223 on IL‐5 and IL‐13, we can infer that miR‐223 promotes inflammation through T2 cytokines. The effect of miRNAs on T2 cytokines in EoE has been previously documented in paediatric EoE cohorts where it was found that miR‐146a and miR‐146b were upregulated in patient serum: both miR‐146a and miR‐146b were found to have a potential role in the promotion of T2 responses through the selective downregulation of T1‐associated genes.^10^ As miR‐223 was found to be upregulated both in serum and oesophageal biopsies,^10^ it is plausible that miR‐223 is manipulating the T helper cell phenotype by exploiting similar mechanisms. Although it should be noted that the upregulation of miR‐223 in oesophageal biopsies has been shown consistently, serum miR‐223 has not always been found to correlate with this.^18^ Future studies will need to elucidate this further.

The transmembrane receptor IGF1R has previously been identified *in vitro* as a target of miR‐223 and confirmed using luciferase assays by multiple research groups.^22^, ^23^, ^24^ Lu *et al*.^25^ previously identified that IGF1R was upregulated in cultured eosinophil progenitor cells from miR‐223^−/−^ mice. It remains untested, but is highly unlikely, that miR‐223 effects on IGF1R can account entirely for the amelioration of all EoE hallmark features observed after miR‐223 inhibition. The lower magnitude of increased miR‐223 expression in the EoE mouse model versus in EoE patients (1.5–2.0 fold, *P = *0.03 versus 10 fold, *P* < 0.001; Figures 1a and 2a) has resulted in a lower magnitude of change in IGF1R expression in the *A. fumigatus* EoE mouse model (Figure 2b) versus in EoE patients (Figure 1b). However, we detected a comparable inverse correlation between miR‐223 and IGF1R expression in *A. fumigatus*‐treated mice (*r* = −0.67, *P = *0.039, Figure 2c) versus EoE patients (*r* = −0.65, *P = *0.0059, Figure 1c).

Causally linking the effects of a specific miRNA on their *in vitro* validated mRNA targets to aspects of disease pathogenesis *in vivo* is challenging. In addition, targeting miR‐223 in a tissue‐specific manner in patients with EoE will require significant efforts and ultimately may be unsuccessful. In contrast, resveratrol has a good safety profile, is readily available, and taken orally may have sufficient local uptake and bioavailability to suppress disease hallmark features in the oesophagus of EoE patients. This is the first demonstration *in vivo* that resveratrol can inhibit MID‐1 in the oesophagus and extends previous *in vitro* studies that identified resveratrol as a direct small molecule inhibitor of MID‐1.^17^ Here, we show for the first time the ability of resveratrol to also regulate downstream miRNA‐223 signalling which is likely to be via MID‐1 inhibition. Resveratrol has also been shown to reduce calpain activity in models of allergic asthma.^26^ Interestingly, calpain 14 is an oesophagus‐specific calpain identified as upregulated in EoE GWAS^27^ and more recently has been shown to play a key role in STAT6‐mediated inflammatory IL‐13 signalling in EoE.^28^ Calpains do not contribute to murine EoE models as shown in mice lack calpain 14.^29^ Thus, the effect of resveratrol reported here is likely to occur independent of modulating calpain activity. Resveratrol's inhibitory action upon calpain activity is complimented by our data showing additional calpain‐independent mechanisms, at a moderate dose range with oral delivery which provide a strong rationale for human trials of resveratrol for the treatment of EoE.

Overall, we report a functional role for miR‐223 in the perpetuation of hallmark EoE features including T2 cytokines and confirm that miR‐223 is a downstream effector of inflammatory MID‐1 signalling in this setting. Resveratrol holds promise to be trialled as a potential future therapeutic intervention in EoE, an increasingly prevalent condition with limited current treatment options.

## Methods

### Patient cohorts and analysis

Patient cohorts and methods for RNA and protein extraction and analysis have been described previously.^30^ In brief, biopsies collected from the distal third of the oesophagus in paediatric patients with active EoE (*n* = 17) and non‐EoE control children (no EoE diagnosis and zero eosinophils per high‐power field, *n* = 21) underwent either RNA or protein extraction. Extracted RNA was utilised in this study for TaqMan™ analysis to determine miR‐223 expression. Written informed consent was obtained from all parents/guardians and assent from children (where age‐appropriate) prior to collection of additional biopsy samples for research purposes during an oesophago‐gastroscopy that was clinically indicated for diagnostic purposes by an appropriately qualified paediatric gastroenterologist. The study was approved by the Hunter New England Local Health District (12/04/18/4.04) and The University of Newcastle Human Research Ethics Committees.

### *Aspergillus fumigatus* model of EoE

Wild‐type (WT) and TRAIL‐deficient (TRAIL^−/−^) BALB/c and WT C57BL/6 mice (male, 8–12 weeks of age) were obtained from Australian BioResources (Moss Vale, NSW, Australia) under a material transfer agreement with Amgen (Macquarie Park, NSW, Australia).^7^ All experiments were approved by the Animal Care and Ethics Committee of the University of Newcastle.

Mice were intranasally challenged with 100 μg of *A. fumigatus* extract (Greer Laboratories, Lenoir, NC, USA) in 50 µL of sterile saline three times a week for three weeks after administration of isoflurane anaesthetic. Control mice received 50 µL of saline only. Oesophageal samples were collected 24 h after the first or final *A. Fumigatus* challenge.

### Antagomir knockdown of miR‐223

Antagomirs targeting miR‐223 and scrambled control antagomirs designed not to target any known miRNA sequence were purchased from Dharmacon (Millennium Science, Mulgrave, VIC, Australia). Mice were administered 50 μg of antagomirs intranasally in 50 µL of sterile saline, 24 h prior to the initial *A. fumigatus* challenged. This dose was repeated daily throughout the course of the model.

### Resveratrol treatment

Resveratrol was administered orally to anaesthetised mice at a dose of 25 mg kg^−1^ suspended in 50 µL with a thickening agent in sterile saline while vehicle controls received thickening agent alone in sterile saline. Drinking water was withheld for 60 min post‐administration. Mice received the initial dose 24 h prior to the initial *A. fumigatus* challenge. This dose was selected based on previous reports of topical application to the lungs in models of allergic airways disease^31^, ^32^ and was repeated daily throughout the course of the model.

### RNA extraction and PCR

Collected oesophageal tissue from mice was immersed in RNA*later®* (Ambion, Life Technologies, Mulgrave, VIC, Australia) prior to being frozen at −80^o^C. RNA was extracted using TRIzol® (Invitrogen, Life Technologies, Mulgrave, VIC, Australia) as per the manufacturer's instructions. miR‐223 expression was quantified from RNA extracted from mouse and human tissue using TaqMan™ primers and chemistry (Life Technologies, Mulgrave, Australia). miR‐223 expression was normalised to U6 in human samples and Sno202 in mouse samples. qPCR was performed using the Eppendorf Realplex PCR system (Hamburg, Germany).

### Protein extraction

Snap‐frozen oesophageal samples underwent protein extraction and were quantified as previously described.^7^, ^30^ Briefly, samples were weighed prior to being homogenised (Tissue‐Tearor, BioSpec Products, Bartlesville, OK, USA) and protein levels for IL‐5, IL‐13, MID‐1 or IGF1R were determined by ELISA (R&D systems, Minneapolis, MN, USAor Cusabio, Wuhan, China). Results are normalised to oesophageal tissue weight.

### Histological analysis of mouse oesophagi

Mid‐sections of oesophageal tissue were fixed in 10% formalin for 24 h before routine processing to paraffin wax, sectioning at 5 μm and underwent H&E staining to enumerate eosinophils. Eosinophil infiltration into the oesophagus was determined by blinded personnel counting the number of eosinophils within 1 mm^2^ of transverse oesophageal section stained with Masson trichrome. The degree of oesophageal fibrosis was determined as the area per micrometer (in μm^2^ per μm) by using Image‐Pro Plus 6 software (Media Cybernetics, Rockville, MD, USA).

### Oesophageal circumference analysis

Oesophageal sections were incised longitudinally and flattened on sections of blotting paper. Photographs were taken of the exercised oesophagi, and oesophageal circumference was measured using Image‐Pro Plus 6 software (Media Cybernetics) as previously described.^7^, ^30^

### Statistical analysis

Statistical significance was determined between experimental groups using ANOVA with Dunnett's correction for multiple testing (Mann–Whitney and Spearman's correlation for human data) in GraphPad Prism 6 (La Jolla, CA, USA). Data are presented as mean ± SEM.

## Conflict of interest

The authors declare no conflict of interest.

## Author Contributions

AMC and JM conceived and designed research. AMC and LAS performed experiments. SN coordinated collection of clinical samples. AMC, LAS, and JM analysed data. AMC, LAS, SN, EP ALF, JM, SK, PSF, and JM interpreted results of experiments. AMC and LAS prepared figures and drafted manuscript. All authors edited and revised manuscript and approved final version.

## References

[1] Dellon ES , Liacouras CA , Molina‐Infante J *et al* Updated international consensus diagnostic criteria for eosinophilic esophagitis: proceedings of the AGREE conference. Gastroenterology 2018; 155: 1022–1033.e1010. 30009819 10.1053/j.gastro.2018.07.009 PMC6174113

[2] Dellon ES , Hirano I . Epidemiology and natural history of eosinophilic esophagitis. Gastroenterology 2018; 154: 319–332.e313. 28774845 10.1053/j.gastro.2017.06.067 PMC5794619

[3] Alexander ES , Martin LJ , Collins MH *et al* Twin and family studies reveal strong environmental and weaker genetic cues explaining heritability of eosinophilic esophagitis. J Allergy Clin Immunol 2014; 134: 1084–1092.e1081. 25258143 10.1016/j.jaci.2014.07.021 PMC4253562

[4] Mishra A , Hogan SP , Brandt EB , Rothenberg ME . An etiological role for aeroallergens and eosinophils in experimental esophagitis. J Clin Invest 2001; 107: 83–90. 11134183 10.1172/JCI10224 PMC198543

[5] Lucendo AJ , Arias A , Gonzalez‐Cervera J *et al* Empiric 6‐food elimination diet induced and maintained prolonged remission in patients with adult eosinophilic esophagitis: a prospective study on the food cause of the disease. J Allergy Clin Immunol 2013; 131: 797–804. 23375693 10.1016/j.jaci.2012.12.664

[6] Gupta SK , Fitzgerald JF , Kondratyuk T , HogenEsch H . Cytokine expression in normal and inflamed esophageal mucosa: a study into the pathogenesis of allergic eosinophilic esophagitis. J Pediatr Gastroenterol Nutr 2006; 42: 22–26. 16385249 10.1097/01.mpg.0000188740.38757.d2

[7] Collison AM , Sokulsky LA , Sherrill JD *et al* TNF‐related apoptosis‐inducing ligand (TRAIL) regulates midline‐1, thymic stromal lymphopoietin, inflammation, and remodeling in experimental eosinophilic esophagitis. J Allergy Clin Immunol 2015; 136: 971–982. 25981737 10.1016/j.jaci.2015.03.031 PMC4600423

[8] Lucendo AJ , Molina‐Infante J , Arias A *et al* Guidelines on eosinophilic esophagitis: evidence‐based statements and recommendations for diagnosis and management in children and adults. United Eur Gasteroenterol J 2017; 5: 335–358. 10.1177/2050640616689525 PMC5415218 28507746

[9] Sherrill JD , Kiran KC , Blanchard C *et al* Analysis and expansion of the eosinophilic esophagitis transcriptome by RNA sequencing. Genes Immun 2014; 15: 361–369. 24920534 10.1038/gene.2014.27 PMC4156528

[10] Lu TX , Sherrill JD , Wen T *et al* MicroRNA signature in patients with eosinophilic esophagitis, reversibility with glucocorticoids, and assessment as disease biomarkers. J Allergy Clin Immunol 2012; 129: 1064–1075.e1069. 22391115 10.1016/j.jaci.2012.01.060 PMC3466056

[11] Sayed D , Abdellatif M . MicroRNAs in development and disease. Physiol Revi 2011; 91: 827–887. 10.1152/physrev.00006.2010 21742789

[12] Pillai RS , Bhattacharyya SN , Artus CG *et al* Inhibition of translational initiation by Let‐7 MicroRNA in human cells. Science 2005; 309: 1573–1576. 16081698 10.1126/science.1115079

[13] Gregory RI , Chendrimada TP , Cooch N , Shiekhattar R . Human RISC couples microRNA biogenesis and posttranscriptional gene silencing. Cell 2005; 123: 631–640. 16271387 10.1016/j.cell.2005.10.022

[14] Collison A , Herbert C , Siegle JS , Mattes J , Foster PS , Kumar RK . Altered expression of microRNA in the airway wall in chronic asthma: miR‐126 as a potential therapeutic target. BMC Pulm Med 2011; 11: 29. 21605405 10.1186/1471-2466-11-29 PMC3116478

[15] Collison A , Mattes J , Plank M , Foster PS . Inhibition of house dust mite‐induced allergic airways disease by antagonism of microRNA‐145 is comparable to glucocorticoid treatment. J Allergy Clin Immunol 2011; 128:160–167.e164. 21571357 10.1016/j.jaci.2011.04.005

[16] Mattes J , Collison A , Plank M , Phipps S , Foster PS . Antagonism of microRNA‐126 suppresses the effector function of TH2 cells and the development of allergic airways disease. Proc Natl Acad Sci USA 2009; 106: 18704–18709. 19843690 10.1073/pnas.0905063106 PMC2773983

[17] Schweiger S , Matthes F , Posey K *et al* Resveratrol induces dephosphorylation of Tau by interfering with the MID1‐PP2A complex. Sci Rep 2017; 7: 13753. 29062069 10.1038/s41598-017-12974-4 PMC5653760

[18] Zahm AM , Menard‐Katcher C , Benitez AJ *et al* Pediatric eosinophilic esophagitis is associated with changes in esophageal microRNAs. Am J Physiol Gastrointest Liver Physiol 2014; 307: G803–G812. 25147232 10.1152/ajpgi.00121.2014 PMC4200319

[19] Collison A , Hatchwell L , Verrills N *et al* The E3 ubiquitin ligase midline 1 promotes allergen and rhinovirus‐induced asthma by inhibiting protein phosphatase 2A activity. Nat Med 2013; 19: 232–237. 23334847 10.1038/nm.3049

[20] Matthaei KI , Foster P , Young IG . The role of interleukin‐5 (IL‐5) *in vivo*: studies with IL‐5 deficient mice. Mem Inst Oswaldo Cruz 1997; 92(Suppl 2): 63–68. 9698917 10.1590/s0074-02761997000800010

[21] Wills‐Karp M , Luyimbazi J , Xu X *et al* Interleukin‐13: central mediator of allergic asthma. Science 1998; 282: 2258–2261. 9856949 10.1126/science.282.5397.2258

[22] Jia CY , Li HH , Zhu XC *et al* MiR‐223 suppresses cell proliferation by targeting IGF‐1R. PLoS One 2011; 6: e27008. 22073238 10.1371/journal.pone.0027008 PMC3206888

[23] Feng SJ , Zhang XQ , Li JT , Dai XM , Zhao F . miRNA‐223 regulates ischemic neuronal injury by targeting the type 1 insulin‐like growth factor receptor (IGF1R). Folia Neuropathol 2018; 56: 49–57. 29663740 10.5114/fn.2018.74659

[24] Yang Q , Xu H , Yang J , Zhou Y , Zhao D , Liu F . MicroRNA‐223 affects IL‐6 secretion in mast cells via the IGF1R/PI3K signaling pathway. Int J Mol Med 2016; 38: 507–512. 27354148 10.3892/ijmm.2016.2649

[25] Lu TX , Lim EJ , Besse JA *et al* MiR‐223 deficiency increases eosinophil progenitor proliferation. J Immunol 2013; 190: 1576–1582. 23325891 10.4049/jimmunol.1202897 PMC3563857

[26] Aich J , Mabalirajan U , Ahmad T *et al* Resveratrol attenuates experimental allergic asthma in mice by restoring inositol polyphosphate 4 phosphatase (INPP4A). Int Immunopharmacol 2012; 14: 438–443. 22986054 10.1016/j.intimp.2012.08.017

[27] Kottyan LC , Davis BP , Sherrill JD *et al* Genome‐wide association analysis of eosinophilic esophagitis provides insight into the tissue specificity of this allergic disease. Nat Genet 2014; 46: 895–900. 25017104 10.1038/ng.3033 PMC4121957

[28] Miller DE , Forney C , Rochman M *et al* Genetic, inflammatory, and epithelial cell differentiation factors control expression of human calpain‐14. G3 (Bethesda) 2019; 9: 729–736. 30626591 10.1534/g3.118.200901 PMC6404614

[29] Litosh VA , Rochman M , Rymer JK , Porollo A , Kottyan LC , Rothenberg ME . Calpain‐14 and its association with eosinophilic esophagitis. J Allergy Clin Immunol 2017; 139: 1762–1771.e1767. 28131390 10.1016/j.jaci.2016.09.027 PMC5461191

[30] Sokulsky LA , Collison AM , Nightingale S *et al* TRAIL deficiency and PP2A activation with Salmeterol ameliorates egg allergen driven eosinophilic esophagitis. Am J Physiol Gastrointest Liver Physiol 2016; 311: G998–G1008. 27742702 10.1152/ajpgi.00151.2016

[31] Chen J , Zhou H , Wang J *et al* Therapeutic effects of resveratrol in a mouse model of HDM‐induced allergic asthma. Int Immunopharmacol 2015; 25: 43–48. 25617148 10.1016/j.intimp.2015.01.013

[32] Royce SG , Dang W , Yuan G *et al* Resveratrol has protective effects against airway remodeling and airway hyperreactivity in a murine model of allergic airways disease. Pathobiol Aging Age Relat Dis 2011; 1: 7134. 10.3402/pba.v1i0.7134 PMC3417665 22953028

